# Polyvinylalcohol Composite Filled with Carbon Dots Produced by Laser Ablation in Liquids

**DOI:** 10.3390/polym16101390

**Published:** 2024-05-13

**Authors:** Mariapompea Cutroneo, Letteria Silipigni, Petr Malinsky, Petr Slepicka, Domenico Franco, Lorenzo Torrisi

**Affiliations:** 1 Department of Physics (MIFT), Messina University, V. le Ferdinando Stagno d’Alcontres 31, S. Agata, 98166 Messina, Italy; lsilipigni@unime.it (L.S.); ltorrisi@unime.it (L.T.); 2 Nuclear Physics Institute, AS CR, 25068 Rez, Czech Republic; pmalinsky@ujf.cas.cz; 3 Department of Physics, Faculty of Science, University of J. E. Purkyně, České mládeže 8, 40096 Ústí nad Labem, Czech Republic; 4 Department of Solid State Engineering, Institute of Chemical Technology, 16628 Prague, Czech Republic; petr.slepicka@vscht.cz; 5 Department of Chemical, Biological, Pharmaceutical Sciences (ChiBioFarAm), University of Messina, V. le Ferdinando Stagno. d’Alcontres 31, S. Agata, 98166 Messina, Italy; domenico.franco@unime.it

**Keywords:** carbon dots, laser ablation in liquid, PVA, photoluminescence

## Abstract

Carbon dots (CDs), owing to their excellent photoluminescent features, have been extensively studied for physics preparation methods and for biomedical and optoelectronic device applications. The assessment of the applicability of CDs in the production of luminescent polymeric composites used in LEDs, displays, sensors, and wearable devices is being pursued. The present study reports on an original, environmentally friendly, and low-cost route for the production of carbon dots with an average size of 4 nm by laser ablation in liquid. Jointly, to prove the significance of the study for a wide range of applications, a free-standing flexible polyvinyl alcohol (PVA) composite containing photoluminescent carbon dots was manufactured. CDs were prepared using targets of porose charcoal with a density of 0.271 g/cm^3^ placed in phosphate-buffered saline (PBS) liquid solution and irradiated for 30 min by pulsed IR diode laser. The optical properties of the obtained suspension containing carbon dots were studied with UV-ViS and FTIR spectroscopies. The photoluminescence of the produced carbon dots was confirmed by the emission peak at 480 nm in the luminescence spectrum. A narrow luminescence band with a full width at half-maximum (FWHM) of less than 40 nm could be an asset in spectral emission analysis in different applications. Atomic force microscopy confirms the feasibility of manufacturing CDs in clean and biocompatible environments, paving the way for an easier and faster production route, crucial for their wider applicability.

## 1. Introduction

Nowadays, smart materials have gathered the attention of the scientific community both for the development of fundamental studies and for commercial applications. The design of new outcomes is critical, as is the functionalization of existing materials for the upgrade of their native features.

The insertion of nanoparticles (either organic or inorganic) into polymers is frequently used as a reinforcement and to promote their flexibility, physical and thermal stability, chemical inertness, higher mechanical properties, and a larger surface area-to-volume ratio.

Pietrzak and Masek [1] reported on the improved photostability of composites filled with carbon black. They observed that after 900 h of exposure to radiation, the aging factor value of the composites was still close to 1.

Rzeczkowski et al. [2] described the increased electrical and thermal conductivities of polypropylene (PP) as a function of graphite fillers with different contents in the range of 10–80 wt%. The effectiveness of the fillers in enhancing the native properties of the matrix depends on their size, shape, aspect ratio, surface area, and dispersion. Conductive fillers, including reduced graphene oxide [3] and carbon nanotubes (CNTs) [4], are purely based on aromatic, non-polar sheets. This means that their interaction with extremely polar molecules, such as water, is very weak, resulting in their immiscibility. The water solubility of carbon dots (CDs), along with their photostability, tunable photoluminescence [5] and biocompatibility, has attracted significant attention in recent years for applications spanning from light emitting diodes (LEDs) [6], solar cells [7], bioimaging [8], and photodetectors [9] to photothermal therapies [10]. Carbon dots are an emerging class of zero-dimensional carbon-based nanoparticles (NPs) with various subgroups based on their crystallinity and morphology.

Typically, nanoparticles can be synthesized with simple and low-cost methods such as electrochemical fabrication [11], solution phase synthesis [7], and laser ablation in liquids [12]. CDs are usually prepared via the hydrothermal carbonization of citric acid functionalized with amine-terminated compounds like ethylenediamine (EDA) [13], branched polyethylenimine (BPEI) [14], and L- cysteine [15] to obtain nitrogen-doped CDs with high photoluminescence quantum yield [13]. The surface functionalities of CDs are influenced by the nature of the source material, environment, solvent, and synthesis process. Miao et al. reported on CD synthesis through the thermal pyrolysis of citric acid and urea, showing the gradual tuning of the maximum emission from blue to red [16]. Qu et al. achieved the production of CDs by a one-step hydrothermal process obtaining a broadband emission over the whole visible wavelength range [17].

Given the strength of reliable results [18] obtained in the preparation of nanoparticles by laser ablation in liquid (PLD), we now emphasize the versatility of this technique as a clean, simple, reproducible, and efficient route for the production of carbon nanodots, i.e., carbon aggregates with a size of the order of a few of nanometers.

Laser ablation could be carried out in different environments, from vacuum [19], air [20], and gases [21] to liquids [22] depending on the desired applications, such as welding [23], medicine [24], basic research [25], and to produce nanoparticles [26].

Kaczmarek et al. [18] described the production of CDs by nanosecond pulsed laser ablation of a graphite target in pure water. The very weak photoluminescence of the thus-produced CDs was increased when they added polyethyleneimine (PEI) [27], which is a cross-linked polymer, to the aqueous suspension. Motivated by the lack of research on producing stable nanoparticles for antibacterial applications, Jonathan et al. [28] reported on the production of carbon NPs using an Nd: YAG laser in different media environments. Nanoparticles produced in chitosan solution displayed greater colloidal stability and a diameter of 38.5 nm. On the other hand, NPs produced in ethylenediamine were less stable but had a diameter of 29.4 nm and improved antibacterial properties, in agreement with the fact that smaller nanoparticles are able to prevent bacterial growth. CDs’ luminescence is due to their functionalization with different molecules that cover the surface of their generally spherical nanostructures. In this sense, molecules containing phosphorus, nitrogen, chlorine, functional oxygen groups, and other species act as a luminescent enhancement.

Although the common critical parameters for laser processing are wavelength, laser fluence, and pulse duration, the environment (air, vacuum, gas) plays a crucial role. It seems that the optimal environment for the production of nanoparticles is liquid [29].

The laser, after the interaction with a solid sample immersed in a liquid, generates a plasma plume. The plasma expands, whereby a vapor layer is formed in the liquid. Next, the vapor layer expands into a cavitation bubble while the plasma shrinks.

The lifetime of the bubble, depending on the laser pulse’s duration and fluence, is crucial for the formation of the structures released into the liquid after the bubble collapses. The dispersed nanostructures keep growing for a short period because the quenching time in liquid is slightly faster than that in air [30]. The produced nanoparticles exhibit high purity as they do not require additional chemical agents during the process.

The literature reports on the production of 100 nm sized CDs by laser ablation in water [31].

The originality of the present study relates to the synthesis of CDs with a size of about 4 nm using a charcoal target laser irradiated in a medium of water and phosphate-buffered saline (PBS). As far as we know, this has never been introduced before, although there are abundant manuscripts in the literature reporting on the production of CDs with sizes larger than 4 nm, using an organic solvent with toxic characteristics, using activators to improve the CDs’ fluorescence properties, and using a more expensive laser system than that used in our research. To explore the applicability of CDs in biomedicine, nanotechnology, and optoelectronic devices, it is beneficial to verify the feasibility of CDs in solid composites [32]. The characteristics of the synthesized suspension of CDs support its embedding in a polymeric matrix of polyvinylalcohol (PVA). This results in the easy and fast processing of nanocomposites with optoelectronic properties, useful for tunable photoluminescence [33], sensors, detectors, and wearable devices.

## 2. Materials and Methods

**Figure 1:**
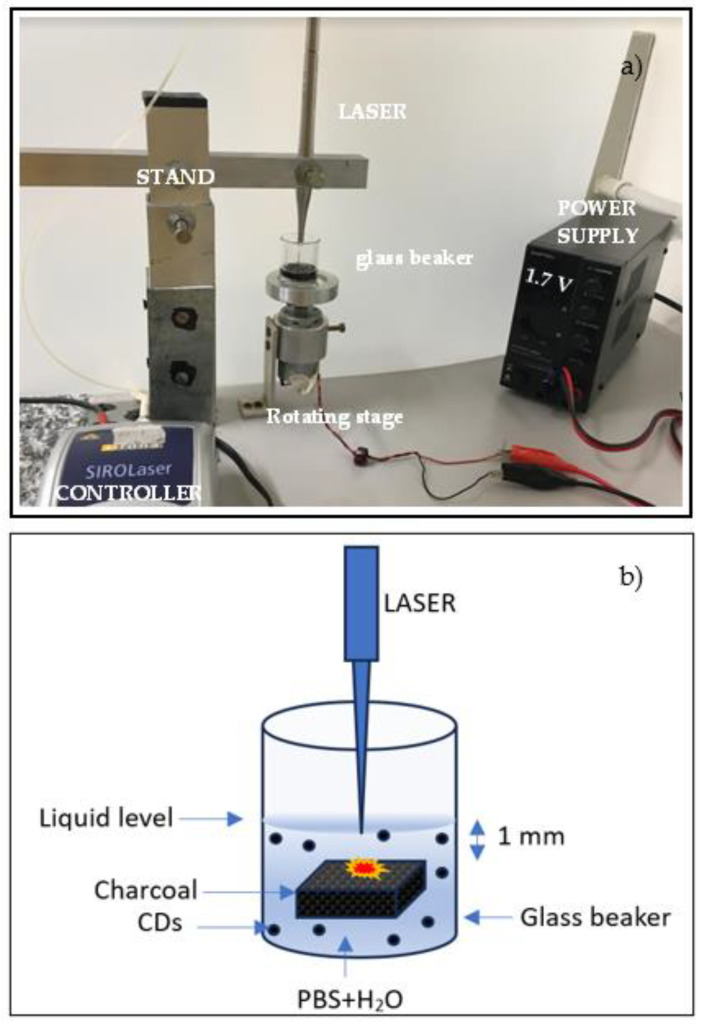
Picture (**a**) and sketch (**b**) of the system for the production of CDs by laser ablation in liquid.

Polyvinylalcohol (PVA) was purchased from R&G Fiber Composite Materials GmbH (GmbH, Waldenbuch, Germany) and used as received. Phosphate-buffered saline (PBS) tablets 2000.0 mg/tab were purchased from Sigma Aldrich (Sigma Aldrich, St. Louis, MO, USA). One tablet of PBS was dissolved in 200 mL of purified water to obtain a balanced salt solution with a pH of 7.2–7.6. This as-prepared isotonic buffer is biocompatible and it is commonly used for diluting and washing cells. In this study, it was used as the liquid for the preparation of CDs by laser ablation and it is labeled as PBS. The synthesis of CDs was accomplished using a Sirolaser diode system (Dentsply Sirona, Charlotte, NC, USA) operating at 970 nm wavelength, 200 ms pulse duration, 7 W power, 750 mJ laser energy, and 10 Hz repetition rate. A picture and a sketch of the adopted experiment setup are shown in Figure 1.

A piece of charcoal was placed in a glass beaker containing 4 mL of PBS solution. Charcoal with low density, high porosity, size of 2.5 cm × 2.5 cm, a thickness of 5 mm, and a weight of 0.848 g was employed as a target during laser ablation in liquid. Charcoal composition depends on the geographical location and on burning parameters such as the temperature and environment [34]. The typical composition of charcoal is 66.9% carbon, 4.4% hydrogen, 7.6% oxygen, 1.3% nitrogen, 1.1% sulfur, 7.2% moisture, 11.5% ash, and 0.1% chlorine. The laser was located on a stand which was movable in the horizontal and vertical directions to allow for the optimization of the distance between the tip of the laser fiber, 200 μm in diameter, and the surface of the charcoal target contained in the glass beaker. This distance was about 1 mm. The beaker was placed on a rotating stage (60 rpm) to ensure the laser irradiation of the immersed surface of the charcoal in different locations. The irradiation time was about 30 min.

Then, the prepared suspension was transferred to a quartz cuvette to be analyzed by UV-VIS spectrometer (Avantes BV, Apeldoorn, the Netherlands) to prove its luminescence.

**Figure 2:**
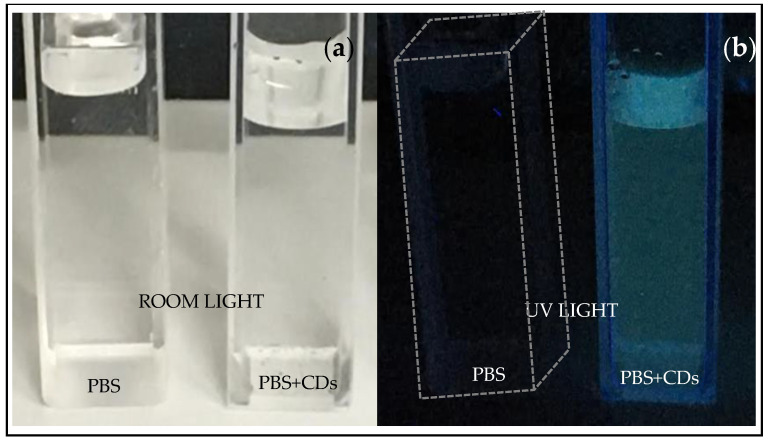
Cuvettes containing PBS and PBS+CDs illuminated by room light (**a**) and illuminated by UV lamp (**b**).

Figure 2 shows cuvettes containing PBS and PBS+CDs, both illuminated by room light (see Figure 2a) and illuminated by UV light at 365 nm wavelength (see Figure 2b). The suspension of PBS+CDs lights up in a blue color under UV light, suggesting emission at a wavelength of 450–495 nm. The cuvette containing PBS is barely perceivable under UV illumination (indeed, it was necessary to draw a dotted white line to mark the edge of the cuvette containing PBS in Figure 2b). Finally, 1.5 mL PVA was mixed with the CDs solutions for 5 min using a magnetic stirrer. The obtained PVA+CDs mixed liquid was poured into a Petri dish, followed by exposure under a Herolab UV lamp (Herolab GmbH Laborgerate, Wiesloch, Germany), operating at 365 nm wavelength for 150 min. The cast films were peeled off from the Petri dish and placed in a desiccator to avoid the effects of water in the air on the hydrophilic PVA. 

A pristine PVA film was obtained by pouring 1.5 mL of PVA into a Petri dish, subsequently exposing it to the UV lamp for 150 min, and then peeling it off from the Petri dish and placing it in a desiccator. The thickness of the PVA+CDs film of about 300 mm was determined by a contact tabletop system.

For morphological analysis of nanoscale structures, atomic force microscopy (AFM) is considered a powerful tool. Its widespread use is attributed to its accurate 2D and 3D reconstruction of sample topography at a relatively low cost and within a short time. AFM was carried out using a Dimension ICON AFM system (Bruker Corp., Bremen, Germany), operating in the PeakForce QNM (Quantitative Nanoscale Mechanical) imaging mode in air. Further equipment includes a commercial silicon tip using the SCANASYST in air mode with a spring constant of 0.4 N/m and a scan size of 3 μm^2^. Drops of the produced CDs solution were placed on silicon wafer cuts and dried in air overnight. Then, the images were processed for the recognition of the CDs using the NanoScope Analysis 1.80 with 32-bit software.

UV–ViS absorption spectra of the suspension and PVA+CDs film were performed using a JASCO-600 spectrophotometer (JASCO, Mary’s Court, Easton, PA, USA) at a working wavelength between 200 nm and 800 nm and a spectral resolution of about 1.0 nm. Attenuated total Reflection Fourier-Transform Infrared (ATR-FTIR) spectra were taken using a JASCO 7600 spectrometer working in the 400–4000 cm^−1^ and spectral resolution of 0.7 cm^−1^.

**Figure 3:**
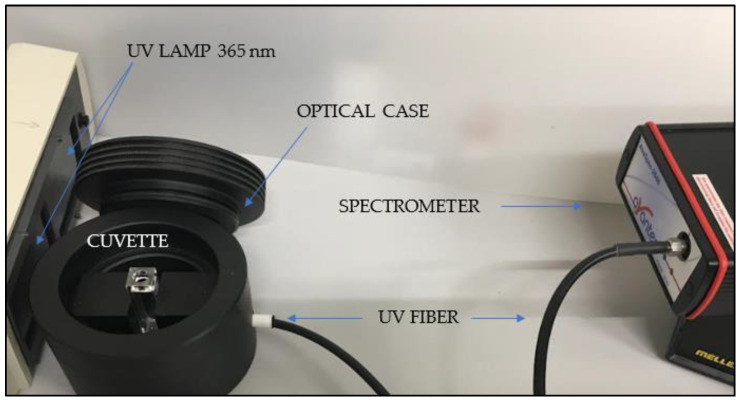
Optical system for luminescence measurements.

The luminescence of PVA+CDs was investigated using an Ava Spec 2048 spectrometer with a spectral resolution of about 1 nm. A case shown in Figure 3 hosts a quartz cuvette illuminated by a Herolab UV lamp operating at a fluence of 37.5 mJ/cm^2^ and a wavelength of 365 nm while a UV fiber connected to the spectrometer is located at 180° with respect to the lamp.

## 3. Results 

**Figure 4:**
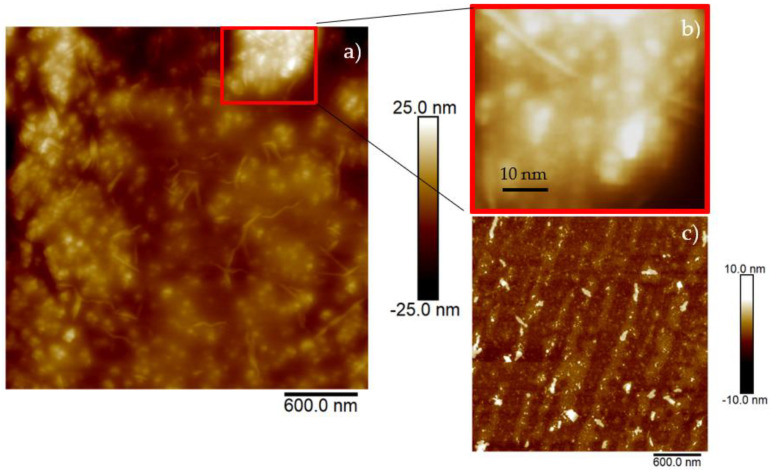
AFM images of silicon wafer surface covered with drops of CDs suspension (**a**), magnified insert (**b**) and pristine silicon wafer surface (**c**).

The surface of the charcoal target used for the production of CDs was about 6.25 cm^2^, with a thickness of 5 mm and a mass of about 0.848 g before the laser irradiation and about 0.835 g after 30 min of laser processing. Assuming all ablated carbon atoms transformed into C-dots, and assuming the size of the CDs was about 10 nm, using the measured density of the used charcoal of about 0.271 g/cm^3^, the number of carbon dots generated by laser ablation should be about 8.8 × 10^16^/cm^3^. Figure 4 presents the image of a cut of a silicon wafer covered with a layer of CDs suspension dried in air (see Figure 4a). The reported size of the structures in the area marked in red is about 4 nm (see Figure 4b). This area is representative of the investigated areas in the whole sample.

**Figure 5:**
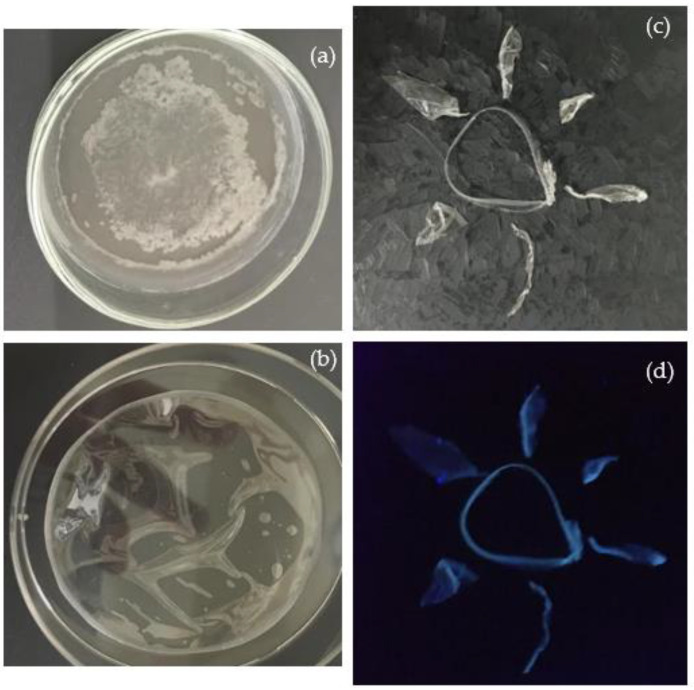
Pristine PVA dried in air without UV lamp (**a**) and assisted by UV lamp (**b**) for 150 min both illuminated by room light; PVA+CDs illuminated by room light (**c**) and by UV light (**d**).

Figure 5 displays PVA foils dried under room light (see Figure 5a) and under UV light at a wavelength of 365 nm (see Figure 5b). PVA is a linear synthetic polymer consisting of a high number of hydroxyl groups in its molecular chains, affecting the chemical and physical properties of PVA. The expected properties of PVA are hydrophilicity, solubility, biocompatibility, non-toxicity, and transparency.

In PVA dried under room light, milky stains are clearly distinguishable in the center and at the edge of the foil, while that dried under UV light appears clear. To preserve the transparency of the manufactured foil, all pristine PVA and the PVA+CDs composites used in this study were dried under UV light. Figure 5c,d) visualize the luminescence of PVA+CDs used to compose a “sun” illuminated by room and UV lights, respectively.

**Figure 6:**
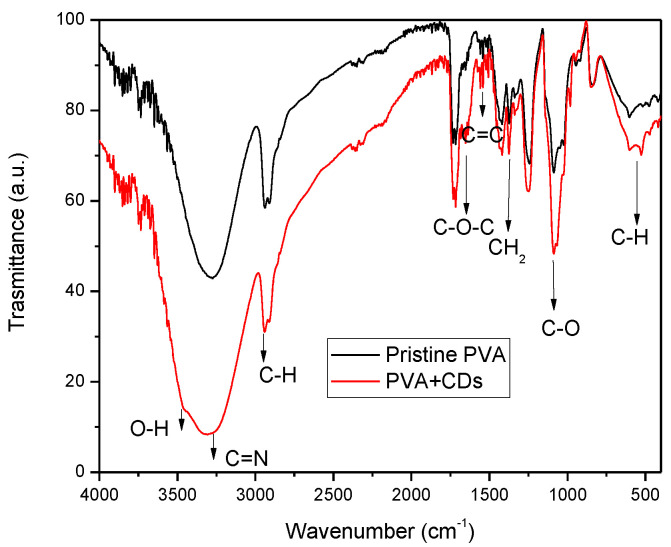
FTIR spectrum for pristine PVA and PVA+CDs foil.

FTIR spectroscopy was used to investigate the chemical structure of CDs. Figure 6 reports broad and merged peaks at around the 2950 and 3384 cm^−1^ bands, attributable to the stretching vibrations of C–H and O–H groups, in agreement with the literature [35]. The bands at 3260 cm^−1^ can be ascribed to the bending vibrations of C=N groups. The presence of the N contribution may be attributable to impurities contained in vegetal carbon.

The peak at 1098 cm^−1^ is representative of the stretching vibrations of C–O. The bands at 1637 cm^−1^ and 1371 cm^−1^ are assigned to the vibrations of C–O–C bonds and CH_2_, while the band at and 1023 cm^−1^ is assigned to C–N. Further absorption bands are displayed in the ranges of 1300–1500 cm^−1^ and 580–780 cm^−1^, corresponding to the bending vibration of C-H from methyl and stretching vibration of C–H from methylene, respectively [36,37].

The results show that the CDs’ surface presented hydrophilic groups, such as hydroxyl, carbonyl and amine groups, stimulating its dispersibility in polar solvents. Further characteristic absorption bands of CDs, like N–H bending vibrations at 1570 cm^−1^, are displayed in the FTIR spectrum. The stretching vibrations of C=C and/or C=N (~1600–1610 cm^−1^) bonds confirm the presence of aggregated carbon or CDs in the analyzed sample [38].

**Figure 7:**
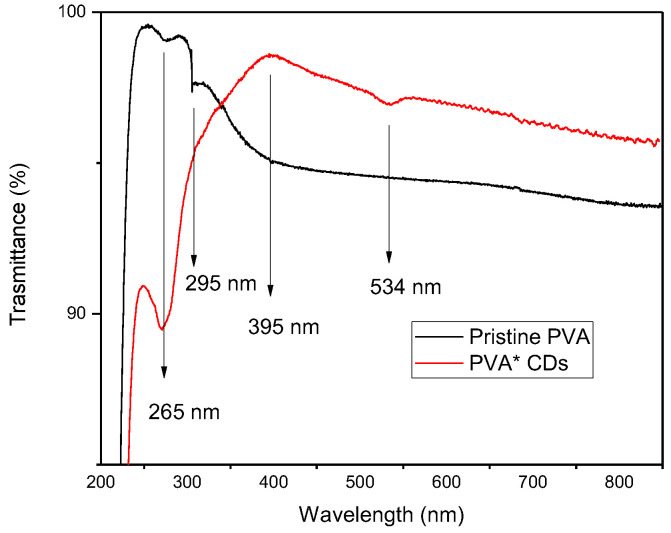
Transmittance on foils of pristine PVA and PVA+CDs.

Figure 7 compares the optical transmittance of pristine PVA and the PVA+CDs composite. The transmittance is complementary to that of the absorption spectrum. PVA is colorless, without any absorption in the visible range. However, the absorption of PVA that can be observed at about 265 nm is assigned to the electronic transitions π→π* of aromatic sp^2^ domains [39] of the conjugated C=C bonds concomitant with a carbon core of CDs and to the n → π* transitions of C=O and COOH groups [35]. The peak around 395 nm could be attributed to the existence of n→π* from C=O.

**Figure 8:**
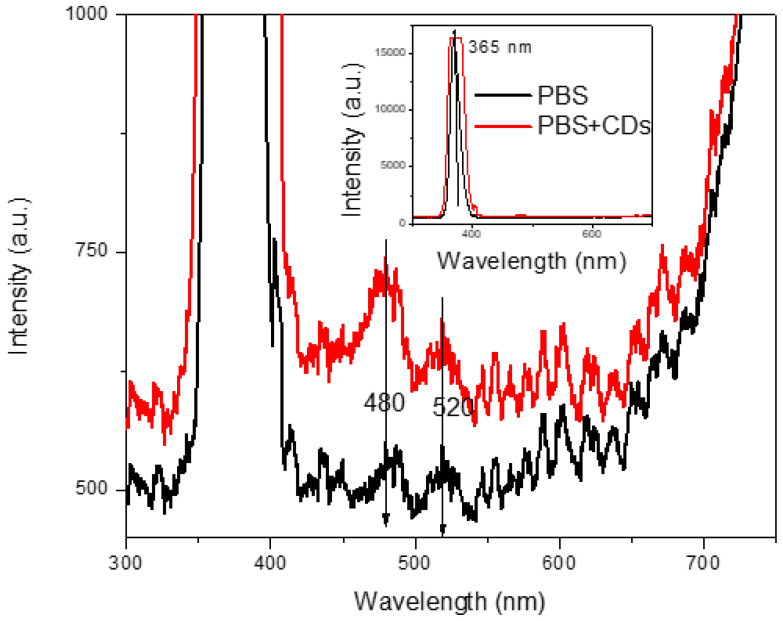
Luminescence of PBS solution and PBS+CDs suspension.

The most fascinating behavior of carbon dots is luminescence under exposure to UV light, as shown in Figure 2. The photoluminescent spectrum displayed in Figure 8 is due to photon excitation occurring around 365 nm (intense peak ranging between 350 nm and 400 nm, see insert of Figure 6) and to the CDs’ light emission at higher wavelengths in the blue and green regions. Our measurements demonstrated that PL intensity is dependent on the concentration of the carbon dots. The intensity of the PL spectra sharply increases with the concentration of carbon dots in the solution. The broad absorption peak lies at about 465 nm, while excitation at the wavelengths of 480 nm and 520 nm. At the excitation wavelength of 465 nm, the suspension mainly emits blue fluorescence around 480 nm, as confirmed in Figure 2. The emission has a broad spectral profile spread over 40 nm.

The photoluminescence of carbon dots can be ascribed to their different sizes and the distribution of different surface electron energy traps of CDs. The variety of sizes of CDs can be confirmed by the different positions of the emission peak in the spectrum. The particles, which are small in size, become excited at a lower wavelength, and larger ones become excited at higher wavelengths [40]. The intensity of the spectrum at 480 nm means that a larger number of particles become excited at that wavelength. In addition, the presence of functional groups on the surface of the carbon dots may induce emissive traps between p and p* of C–C. Indeed, as confirmed by Bibekanada et al. [41], when carbon dots are illuminated at a specific wavelength, a surface energy trap dominates the emission. This plays a significant role in photoconduction, luminescence, and the working system of electronic devices because the ability of a solid semiconductor to carry an electrical current depends on the flow of electrons and holes through the solid.

The compatibility of the produced CDs with polymeric matrices enables their embedding in PVA. Both materials, the CDs and the PBS and PBA solutions used, are biocompatible and can be employed for applications in biological environments.

This can assist with the applicability of CDs in more fields. Considering the enhancement of surface passivation for the carbon dots in the PVA matrix, which is a confined environment, this may result in the improvement of fluorescence emissions [42].

## 4. Discussion

The literature reports that the source of the photoluminescence of carbon nanodots can be attributed to the doping of carbon nanoparticles with oxygen and nitrogen heteroatoms [13,43]. Typically, different emission wavelengths in the solution can be ascribed to the varying conjugation degree at different pH values influencing its functionalities, such as carboxyl and amino during the carbonization stage [44]. It is well established that the surface functional groups of CDs depend on the oxidation of precursors and on their composition. In optical UV-Vis spectroscopy, absorption peaks in the ultra-violet (UV) region below 280 nm could be attributable to π-π* transitions of the C=C bonds within conjugated fragments in the carbon core [45] and to the n → π* transitions of C=O and COOH groups, in agreement with the literature [35]. 

Moreover, FTIR analysis confirmed that CDs contain core and functional groups on their surface, like hydroxyl groups (–OH), carboxyl groups (–COOH), carbonyl groups (–CO), and amino groups (–NH_2_) [46].

The crucial characteristic of CDs is the changing of the emission at specific wavelengths, typically termed excitation-dependent emission. This is due to the presence of various electron orbitals in CDs, resulting in a change in emission regardless of the energy absorbed by the CDs. Emission variations depend on several factors, including the structure of the formed CDs and the presence of defective traps due to oxygenated groups [47].

The emission peak observed in the luminescence spectrum, at 480 nm (blue light), confirms the production of CDs by laser ablation in PBS as qualitatively visualized in the cuvette illuminated by UV light (Figure 2). Further steps in this study were the manufacturing of a PVA foil containing CDs to support their possible applications as sensors and wearable devices.

Indeed, the reported CDs, absorbing light in the UV region and emitting it in the visible region, resulting in strong tissue absorption and superficial tissue penetration, could be promising for in vivo bioimaging and turn-on fluorescent nanosensors.

The blue shift in the emission of the synthesized CDs, which is probably due to self-oxidation in the presence of oxygen, could be further studied for the manufacturing of white-emitting diodes without using additional luminescent materials such as rare earth metals [48].

## 5. Conclusions

This work presents a new route for the synthesis of CDs from raw charcoal irradiated by laser in PBS without the use of external heteroatoms such as sulfur and nitrogen to dope the CDs.

AFM analysis indicated the presence of spherical structures with a size of 4 nm. Optical analysis confirmed the luminescence of CDs, indicated by an increase in the emission peak at 480 nm under an excitation light of 365 nm. To better investigate the potential of the produced CDs, PVA foils containing CDs were produced and characterized by UV-VIS and FTIR.

The results revealed that the PVA+CDs film had good optical properties, which could effectively reduce UV by absorbing short wavelengths.

Emission peaks around 450 nm–490 nm confirmed the luminescence of the PBS+CDs suspension in the blue wavelength, as suggested by the lighting up of the cuvette containing the suspension when illuminated with a UV lamp.

Although many promising results have recently been reported for PVA nanocomposite, none are related to the use of CDs produced by laser irradiation of charcoal in a liquid of PBS. Typically, the filtration and purification of suspensions containing CDs are necessary post-treatments in synthesis of the obtained CDs to promote their incorporation in matrices or their non-toxicity in biological applications. However, the final filtration product is never composed of sole CDs. Studies on producing stable nanoparticles for antibacterial applications, as well as on the effect of liquid environments on the synthesis of CDs, are still limited.

Topics related to the production and characterization of CDs are very prospective research areas. Despite great efforts toward the manufacturing of CDs and the development of composites containing CDs, their functionalization mechanism is not fully understood, and the effect of different liquid environments on their synthesis is still insufficiently described. However, their biocompatibility, size of less than 10 nm, fluorescent properties, low toxicity, and many types of functional groups (amino, hydroxyl, and carboxyl compounds) make CDs promising for a broad range of applications in photocatalysis, bioimaging, photonics, drug delivery systems, or materials science. The present study offers a way to produce CDs without the use of activators and post-treatments such as filtration or purifications. The size of the CDs is about 4 nm, which is suitable for antibacterial applications as well as in drug delivery systems. Possible applications include the use of CDs in biomedicine due to the available preparation of PVA films containing CDs, which could be further investigated for tissue engineering, shape memory material, and biosensors.

## Data Availability

Data are contained within the article.
